# Utility of Magnetic Resonance Spectroscopy and Perfusion Imaging in Differentiating Brain Tumors From Mimics in a Tertiary Hospital in the Philippines

**DOI:** 10.7759/cureus.81258

**Published:** 2025-03-26

**Authors:** Alyssa Pauline C Co, Maria Angelica Liza Imperial, Anna Theresa Dantes, Ron Pilotin, Ranhel De Roxas-Bernardino, Paul Vincent Opinaldo, Julette Marie F Batara

**Affiliations:** 1 Neurology, St. Luke's Medical Center, Quezon City, PHL; 2 Neuro-Oncology, St. Luke's Medical Center, Quezon City, PHL; 3 Neuroradiology, St. Luke's Medical Center, Quezon City, PHL

**Keywords:** brain tumor, glioma, mri, mrs, radiation necrosis

## Abstract

Background: A wide range of non-neoplastic brain lesions can mimic tumors on magnetic resonance imaging (MRI), creating diagnostic challenges. Although MRI is the gold standard for evaluating brain lesions, differentiating between neoplastic and non-neoplastic lesions, as well as high- and low-grade tumors, can be difficult, sometimes leading to unnecessary biopsies. Magnetic resonance spectroscopy (MRS) helps by analyzing biochemical and metabolic processes, especially when conventional MRI falls short. Perfusion MRI (MRP), sensitive to microvasculature, is used to classify tumors, detect strokes, and evaluate other conditions. Both methods are non-invasive alternatives to radiation-based imaging techniques.

Methods: A retrospective, cross-sectional study of patients with intracranial lesions who underwent MRS and perfusion imaging in a tertiary hospital in the Philippines were analyzed.

Results: The study included 37 patients (28 male, 9 female) aged 19-78, with MRS and MRP data collected. Seizures were the most common symptom (24%), followed by weakness, headache, dizziness, and visual changes. Notably, 24% were asymptomatic. Among all patients examined by MRI with MRS and MRP for intracranial mass lesions, 60% were neoplasm, 21.6% were radiation necrosis, 5.4% were demyelinating lesions, 2.7% were infection, and 2.7% were vascular lesions. Biopsies were performed on nine patients, with seven correlating to MR results. Thirty-five point one percent of patients showed no clinical change, while 18.9% fully recovered. Imaging revealed lesion reduction in 35.1% of patients, no change in 29.7%, and lesion growth in 18.9%.

Conclusion: MRS and MRP complement conventional MRI in distinguishing neoplastic from non-neoplastic lesions, differentiation of types of malignancies, and differentiating tumor recurrence from radiation necrosis, offering a non-invasive way of catechizing the biochemical make-up of intracranial lesions.

## Introduction

The imaging and clinical management of patients with CNS neoplasms continue to evolve over time and now heavily rely on physiologic imaging in addition to high-resolution structural imaging [1]. Imaging remains a powerful and useful non-invasive tool that aids in the diagnosis and management of patients with lesions in the brain. Non-invasive neuroimaging techniques offer the opportunity to incorporate functional, hemodynamic, metabolic, cellular, microstructural, and genetic information into the assessment of CNS lesions [1-2]. Specifically, these imaging tools are being applied to diagnose and grade CNS neoplasms preoperatively, plan and navigate surgery intraoperatively, monitor and assess treatment response and patient prognosis, and understand the effects of treatment on the patient’s brain [2].

Magnetic resonance imaging (MRI) offers a non-invasive approach for diagnosis, grading, and post-treatment follow-up of cranial neoplasms. Despite the fact that it is considered the gold standard for the evaluation of brain lesions, there are a lot of instances where a reliable differentiation of neoplastic from non-neoplastic brain lesions or of high- from low-grade tumors is difficult with conventional MRI, which often leads to misdiagnosis. As a result, unnecessary brain biopsies may be performed on patients with non-neoplastic lesions [2-3]. Hence, accurate diagnosis is vital in the choice of treatment and determining the optimal treatment plan.

Magnetic resonance spectroscopy (MRS) can assess neurological abnormalities at the microscopic level by quantifying cellular metabolites and studying their distribution in the tissues. It is extremely useful when conventional MRI fails to resolve certain clinical questions. Differentiating neoplasms from infectious processes and post-radiation effects is often difficult. MRS offers a non-invasive means of assessing the biochemical and metabolic processes in intracranial tissues without the ionizing radiation associated with both positron emission tomography and single-photon emission computed tomography imaging. The social value of using MRS in brain tumors lies in its ability to improve diagnosis, treatment planning, patient outcomes, and research, while reducing the need for invasive procedures and healthcare costs [2-4].

Perfusion MRI (MRP) provides information about tissue vascularization, in vivo tumor angioneogenesis, and microcirculation. MRP offers information about tissue blood volume, blood flow, and oxygenation of tissues. The two main methods of MRP imaging include T2-weighted dynamic susceptibility contrast-enhanced (DSC) perfusion and T1-weighted dynamic contrast-enhanced (DCE) perfusion. DSC is a first-pass bolus tracking blood volume technique, and DCE is a steady-state permeability technique. Both can be used to derive multiple perfusion parameters such as cerebral blood volume (CBV) and endothelial transfer coefficient (Ktrans) [3]. Relative cerebral blood volume (rCBV), which is the calculated CBV relative to the contralateral side, is the most widely used parameter derived from DSC and is considered a marker of angiogenesis. rCBV may be helpful in distinguishing high-grade from low-grade gliomas, as high-grade gliomas have been found to have higher rCBV than low-grade gliomas; however, this should be used with caution, as oligodendrogliomas can have high rCBVs [3-6].

This study aims to evaluate the utility of MRS and perfusion imaging in characterizing brain lesions in the Philippine setting, specifically in cases where conventional MRI alone is insufficient for differentiation. Additionally, it seeks to provide further evidence to support the existing body of knowledge, demonstrating that these advanced imaging techniques can reduce the need for unnecessary invasive diagnostic procedures, such as biopsy.

## Materials and methods

This is a retrospective, cross-sectional study using data from patients with cerebral lesions who underwent MRS and perfusion imaging from 2015 to 2023 in a tertiary hospital in the Philippines.

A list of patients who underwent MRS and perfusion imaging was obtained from the hospital's computerized database and online imaging portal. Data was collected through a review of medical records and the healthcare system. Information regarding the outcomes of patients who received MRS/MRP imaging was recorded in the researchers’ database, which included demographic profiles, comorbidities, neurological symptoms, histopathological findings, clinical diagnoses, management strategies, interventions, and clinical outcomes.

Descriptive statistics were employed to summarize the demographic and clinical profiles of the patients. The findings from MRS/MRP imaging were compared with the final diagnoses and histopathological results when available. A thorough chart review was conducted for all patients included in this study. There was no direct interaction between the researchers and the patients, and no identifying information was included in the data collection form to maintain confidentiality.

Magnetic resonance imaging

All studies were done using a 3.0 Tesla MR scanner (Philips Achieva, Philips Healthcare, USA). Conventional sequences included axial T1-weighted, T2-weighted, fluid-attenuated inversion recovery (FLAIR), diffusion-weighted imaging (DWI), apparent diffusion coefficient (ADC), gradient echo (GRE), sagittal T2, axial post-contrast FLAIR, and post-contrast T1 in three orthogonal planes.

Magnetic resonance perfusion

Perfusion imaging was performed using both DCE-MRI and DSC-MRI, covering the entire brain. DCE was performed first, serving as a preloading dose for the second injection, whereby DSC images were acquired. Gadolinium contrast was administered through an intravenous access in the antecubital vein with a dose of 0.1 mmol/kg of body weight. A rate of 3 cc/second was used for DCE, while a rate of 5 cc/second was used for DSC. Each contrast injection was followed by a saline bolus. Post-processing was performed using the Philips IntelliSpace Portal application.

Magnetic resonance spectroscopy

In all cases, single-voxel and multi-voxel proton MRS (1H-MRS) were performed. For single voxel spectroscopy, a short echo time (TE) of 35 and an intermediate TE of 144 were used, with a voxel size of 1.5 cm³ to 2.0 cm³. The voxel was placed in the optimal location that includes the most volume of the lesion, avoiding areas of necrosis, hemorrhage, and calcifications, as well as minimizing contamination from surrounding tissues. For multivoxel spectroscopy, an intermediate TE of 144 was used. Spectrum analysis was performed using the Philips IntelliSpace Portal application.

Ethical consideration

The research received formal approval (RPC-088-03-24) from the St. Luke's Medical Center Institutional Ethics Review Committee, ensuring compliance with ethical standards. The study adhered to the Declaration of Helsinki (2013), which outlines ethical principles for medical research involving human subjects, and followed the International Council for Harmonization - Good Clinical Practice (ICH-GCP) guidelines, ensuring that the rights, safety, and well-being of participants were prioritized throughout the research process. Data privacy was strictly observed in accordance with the Data Privacy Act, with all personal information anonymized and stored securely to protect participant confidentiality. Access to data was restricted to authorized personnel, and the data was used solely for the purposes of this research.

## Results

A total of 37 patients were included in the study, and MRS and perfusion data were obtained. The age range of patients was from 19 to 78 years. Out of 37, 28 patients were male and 9 were female.

The most common presenting symptom was seizure, seen in 24% of the patients, followed by weakness (16%), headache (11%), dizziness (8%), and visual changes (7%). Other complaints were craniopathies, memory lapses, and a decrease in sensorium and fever. There were also 24% of the patients who were asymptomatic. The majority of the patients (32%) had no known comorbidities, while 27% of the patients had cancer, the majority of which were breast cancer (Table 1).

**Table 1:** Baseline demographics and clinical profile of patients (n = 37)

Parameters	Values (n = 37)
Age in years, standard deviation	50.9 + 14.9
Male: Female	28:9
Neurologic symptom	
Seizure	9
Asymptomatic	9
Weakness	6
Headache	4
Dizziness	3
Visual changes	2
Craniopathies	1
Memory lapses	1
Decrease in sensorium	1
Fever	1
Comorbidities	
None	12
Hypertension	7
Breast cancer	6
Lung cancer	2
Diabetes	2
Recurrent astrocytoma	2
Renal cancer	1
Thyroid cancer	1
Glioblastoma	1
Glioma	1
Osteoarthritis	1
Dyslipidemia	1

Table 2 shows the distribution of lesions/results of MRS/MRP. The most common cause of intracranial space-occupying lesions detected in the study was metastasis, constituting 24.3% of the cases. This was followed by radiation necrosis (21.6%), glioma (18.9%), glioblastoma (8.1%), primary central nervous system lymphoma (PCNSL) (5.41%), pseudoprogression (5.41%), and demyelination (5.41%). Other lesions include anaplastic astrocytoma, choroid papilloma, infection, and vascular.

**Table 2:** Distribution of lesions

Lesion	Number, total n = 37	Percentage
Metastasis	9	24.30
Radiation necrosis	8	21.60
Glioma	7	18.90
Glioblastoma	3	8.10
Primary CNS lymphoma	2	5.41
Pseudo progression/treatment-related changes	2	5.41
Demyelination	2	5.41
Anaplastic astrocytoma	1	2.70
Choroid papilloma	1	2.70
Infection	1	2.70
Vascular	1	2.70

**Figure 1:**
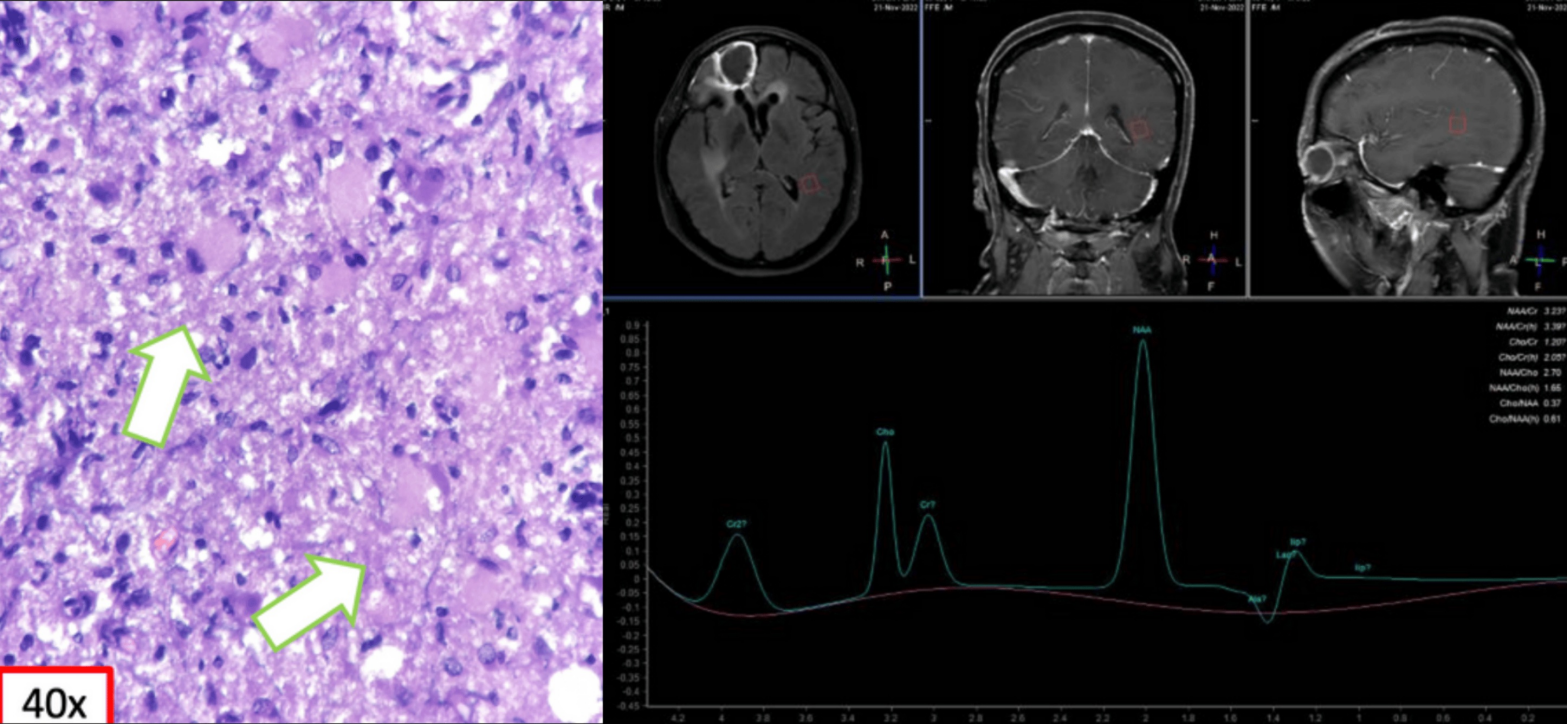
MRS of a 45/F presenting with confusion showed choline and lipid/lactate peaks with a corresponding decrease in NAA suggestive of neoplasm. However, biopsy revealed histiocytic and lymphocytic aggregates in the brain parenchyma, and the leptomeninges may represent changes attributable to radiotherapy (arrows) MRS: magnetic resonance spectroscopy; NAA: N-acetyl aspartate

Table 3 summarizes the MRS/MRP imaging, interventions, clinical outcomes, and post-intervention imaging results of all patients. Among 37 patients, nine (24.3%) underwent biopsy, and seven of these cases showed correlation with MRS and MRP results. The two patients with incongruity of results between conventional MRI and MRS/MRP findings were patient no. 27 with an imaging diagnosis of low-grade glioma, but biopsy revealed post-radiation necrosis (Table 3, Figure 1), and patient no. 30 with an imaging diagnosis of CNS lymphoma, but biopsy revealed demyelination (Table 3). For the remaining patients who did not undergo biopsy, the MRS/MRP results were clinically compatible with the radiologic diagnosis. All patients were treated according to their final diagnosis. Clinically, 35.1% of patients had no clinical change, 18.9% experienced complete resolution of symptoms, 10.8% showed clinical improvement, 8.1% (n = 3) expired, 5.4% had recurrence of symptoms, and 2.7% showed progression of symptoms. On repeat imaging with conventional MRI, the majority of patients (35.1%) showed decreased lesion size and/or edema, 29.7% had no significant change, 18.9% exhibited an increase in size and/or edema, and 5.4% developed a new lesion.

**Table 3:** MRS/MRP imaging, interventions, clinical outcomes and post-intervention imaging (MRI) CNS: central nervous system; IDH: isocitrate dehydrogenase; IMRT: intensity-modulated radiation therapy; MRS: magnetic resonance spectroscopy; MRP: magnetic resonance perfusion; PCNSL: primary central nervous system lymphoma; RT: radiation therapy; SRS: stereotactic radiosurgery; WBRT: whole brain radiation therapy

Patient n = 37	MRS/MRP result	Biopsy	Histopathology	Interventions done	Clinical outcome	Repeat imaging result
1	Demyelination	NA	NA	NA	NA	NA
2	Neoplasm: Metastasis	NA	NA	SRS, targeted therapy (nimotuzumab and bevacizumab)	Complete resolution	No significant change
3	PCNSL vs. high-grade glioma	NA	NA	NA	Lost to follow-up	NA
4	Choroid papilloma	NA	NA	SRS	Complete resolution	Decrease in size and/or edema
5	Anaplastic astrocytoma	Yes	Glioblastoma grade 4	IMRT + concurrent chemotherapy (temozolomide), adjuvant chemotherapy (temozolomide), bevacizumab	Last follow-up 5 months post-biopsy	Increase in size and/or edema, Appearance of new lesion
6	Pseudoprogression	Yes	Glioblastoma, IDH wild type	IMRT + concurrent chemotherapy (temozolomide), adjuvant chemotherapy (temozolomide), bevacizumab	Expired 1 month post MRS/MRP	No repeat MRI
7	Neoplasm: Metastasis	NA	NA	SRS	Complete resolution	Decrease in size and/or edema
8	Neoplasm: Metastasis	NA	NA	SRS	Complete resolution	Decrease in size and/or edema
9	Neoplasm: Metastasis	NA	NA	WBRT	Complete resolution	Decrease in size and/or edema
10	Low-grade glioma	NA	NA	SRS	No clinical change	No significant change
11	Post-radiation necrosis	NA	NA	SRS	No clinical change	Increase in size and/or edema
12	Neoplasm: Metastasis	NA	NA	SRS	Complete resolution	No significant change
13	Vascular	NA	NA	NA	NA	NA
14	Glioblastoma	Yes	Glioblastoma	SRS, chemotherapy (temozolamide), left temporal craniotomy, and excision of tumor	No clinical change	No significant change
15	Glioblastoma	Yes	Tumor recurrence admixed with treatment-related necrosis	Bevacizumab	Lost to follow-up 4 months post-treatment	Increase in size and/or edema, appearance of new lesion
16	Low-grade glioma	Yes	Astrocytoma	Craniotomy, excision of tumor, right parietal area, chemotherapy (temozolomide)	Complete resolution	Decrease in size and/or edema
17	Infection	NA	NA	NA	NA	NA
18	Neoplasm: Metastasis	NA	NA	SRS	No clinical change	No significant change
19	Radiation necrosis	NA	NA	Monitoring	No clinical change	No significant change
20	Residual tumor	NA	NA	Monitoring	No clinical changes	No significant change
21	Radiation necrosis	NA	NA	Bevacizumab infusion	Recurrence after 13 months	Decrease in size and/or edema
22	Neoplasm	NA	NA	Chemotherapy with temozolomide	No clinical change	No significant change
23	CNS lymphoma vs. demyelinating disease	NA	NA	Therapeutic trial with rituximab for 8 doses and dexamethasone	Lost to follow-up	NA
24	Neoplasm	Yes	Glioblastoma, IDH wild type	RT + Chemotherapy (temozolomide), adjuvant temozolomide, targeted therapy with bevacizumab and nimotuzumab	Expired 1 year post biopsy, recurrence at 3rd month post biopsy	Increase in size and/or edema, appearance of new lesion
25	Low-grade glioma	NA	NA	Lost to follow-up	NA	NA
26	Neoplasm: Metastasis	NA	NA	WBRT	Clinically improving	Decrease in size and/or edema
27	Low-grade glioma	yes	Treatment-related necrosis	Continue adjuvant chemotherapy temozolomide	Clinically improving	Decrease in size and/or edema
28	Radiation necrosis	NA	NA	Bevacizumab infusion	No clinical changes	Decrease in size and/or edema
29	Radiation necrosis	Yes	Radiation necrosis	Continue adjuvant chemotherapy temozolomide	Clinically improving	Decrease in size and/or edema
30	CNS lymphoma	Yes	Demyelination	Rituximab infusion	Clinically improving	Decrease in size and/or edema
31	Radiation necrosis	NA	NA	Continue adjuvant metronomic Temozolomide	No clinical changes	Decrease in size and edema
32	Glioma	NA	NA	Supportive care, dexamethasone	No clinical changes	No significant changes in size
33	Glioblastoma	no	NA	Metronomic temozolomide, bevacizumab and nimotuzumab	Further progression, expired after 6 months	Increase in tumor bulk
34	Radiation necrosis	No	NA	Dexamethasone	No clinical changes	Increase in size on 5th month post diagnosis
35	CNS lymphoma	NA	NA	RT+ chemotherapy	No clinical changes	No significant change
36	Radiation necrosis	NA	NA	Metronomic temozolomide	No clinical changes	No significant change
37	Glioma	NA	NA	No treatment	Lost to follow-up	NA

## Discussion

Neuro-oncologists often encounter significant diagnostic challenges when a patient's clinical and radiographic findings suggest multiple potential causes. Accurate diagnosis is critical, as treatment plans must be tailored to the underlying condition, and any delays in diagnosis can worsen the patient's prognosis. When conventional neuroimaging fails to provide a definitive diagnosis, and brain biopsy is not a viable option, advanced imaging techniques like MRS and MRP can offer valuable supplementary information [5-6]. These modalities provide detailed tissue characterization that aligns closely with histological and biochemical findings, helping to narrow down differential diagnoses. Since their introduction, numerous studies have demonstrated the utility of MRS and MRP in diagnostic radiology [7].

Our study showed that for patients selected to undergo MRS and MRP due to inconclusive findings on conventional MRI, these advanced techniques significantly reduced the number of differential diagnoses, often leading to a single, definitive diagnosis. MRS and MRP are particularly valuable in distinguishing recurrent tumors from radiation necrosis, a common challenge in post-treatment monitoring. By providing detailed metabolic and perfusion information, these techniques enable clinicians to make more accurate diagnostic and therapeutic decisions.

The integration of MRS and MRP into patient care helped physicians develop individualized treatment plans, with patient outcomes varying based on the complexity of their conditions. While all patients showed some degree of improvement, the extent of their responses varied, likely due to differences in underlying pathologies and patient-specific factors.

MRS and MRP are advanced imaging modalities that significantly enhance the sensitivity of detecting malignant lesions compared to conventional imaging methods. MRS analyzes specific metabolite levels, such as N-acetyl aspartate (NAA), a neuronal marker often reduced in tumors, particularly high-grade gliomas; choline (Cho), a marker for cellular membrane turnover that is typically elevated in neoplasms; and creatine (Cr), which reflects cell membrane turnover and is often elevated in tumors and other pathological conditions [4-6]. This analysis helps differentiate between benign and malignant lesions. MRP evaluates perfusion characteristics, including cerebral blood flow (CBF) and rCBV, which can indicate malignancy [5]. These modalities are particularly useful for the early detection of tumors, allowing for timely interventions. Additionally, MRS and MRP are crucial in distinguishing recurrent tumor growth from radiation necrosis. MRS identifies abnormal metabolic profiles associated with cellular activity, while MRP assesses blood flow patterns - radiation necrosis typically exhibits lower perfusion compared to active malignancy [5-7].

MRS also tracks metabolic changes over time, offering insights into tumor response to treatment, while MRP monitors shifts in blood flow dynamics, providing early indications of treatment efficacy or disease progression, often before structural changes become visible on conventional imaging. By integrating MRS and MRP with conventional MRI sequences, a multiparametric approach is created, enhancing diagnostic accuracy through a comprehensive evaluation of both the structural and functional characteristics of lesions [8]. This approach is critical for accurate diagnosis, treatment planning, and ongoing follow-up, particularly in complex cases such as differentiating tumor recurrence from treatment-induced changes. Furthermore, MRS and MRP are invaluable in diagnosing inoperable malignant midline brain lesions like diffuse pontine gliomas, where early and accurate diagnosis significantly improves survival rates.

Magnetic resonance spectroscopy and magnetic resonance perfusion: parameters and diagnostic features

**Figure 2:**
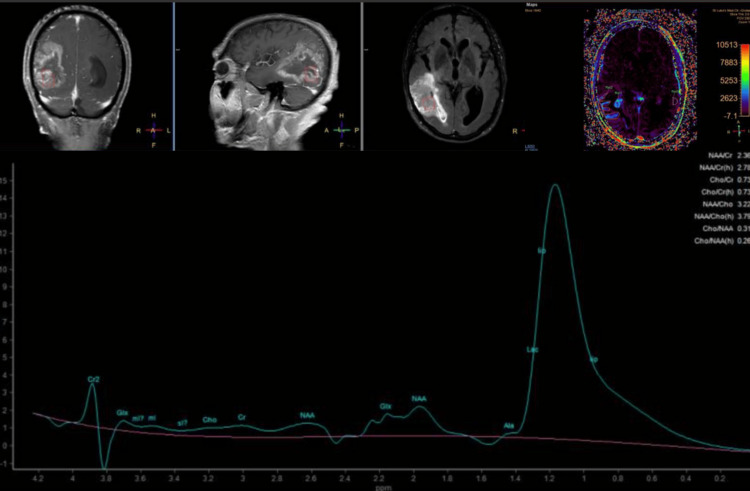
79/M, presenting with headache. MRS showed mild elevation of choline metabolite relative to the creatine and NAA peaks, with significant elevation of the lipid-lactate peak at the lower border of the mass. MRP showed a modest elevation of the relative cerebral blood volume (rCBV). Imaging findings align with the clinical diagnosis of glioblastoma MRS: magnetic resonance spectroscopy; NAA: N-acetyl aspartate; MRP: magnetic resonance perfusion

MRS works by detecting different metabolites within tissues based on their magnetic properties when exposed to a strong magnetic field. Each metabolite resonates at distinct frequencies, a phenomenon known as chemical shift. By quantifying metabolites such as NAA, Cho, and Cr, MRS offers biochemical insights into tissue composition. Elevated choline levels and reduced NAA are commonly associated with malignancy, while increased Cho/Cr ratios often suggest high cellular turnover, a hallmark of neoplastic activity. In contrast, benign lesions often show more balanced metabolite profiles (Figure 2) [9-10].

MRP, on the other hand, assesses tissue blood flow dynamics by tracking contrast agents' distribution and concentration over time. Malignant tumors typically show altered vascularity, such as increased rCBV or abnormal perfusion patterns, compared to normal tissues. MRP’s ability to measure CBF and mean transit time (MTT) enables the differentiation of tumor types and helps evaluate tumor aggressiveness. The lower perfusion observed in radiation necrosis, in contrast to the higher perfusion in active tumors, provides crucial insights in post-treatment monitoring [5,9-10].

Study strengths and limitations

This study provides valuable insights into the use of MRS and MRP in the field of neuro-oncology in the Philippines, where these techniques are not yet widely adopted. As the first study in the country to explore their use, it offers a pioneering approach to improving diagnostic accuracy and treatment planning. The use of MRS and MRP significantly reduced the differential diagnoses, often leading to a single, definitive diagnosis and enhancing diagnostic confidence in cases where conventional MRI alone was inconclusive. These techniques proved particularly effective in differentiating recurrent tumors from radiation necrosis, a critical challenge in post-treatment monitoring.

Additionally, our study utilized a multiparametric imaging approach, integrating MRS and MRP with conventional MRI to provide a comprehensive evaluation of both structural and functional tumor characteristics. The ability to monitor metabolic and perfusion changes over time facilitates early detection of tumor recurrence and treatment efficacy, often before conventional imaging detects structural changes. This integration allowed for more personalized treatment planning, as clinicians could tailor interventions based on each patient's unique condition. This is especially important for complex or inoperable cases, such as malignant midline brain lesions like diffuse pontine gliomas, where early diagnosis is crucial for improving survival outcomes.

However, several limitations must be considered. The relatively small sample size may limit the generalizability of our findings to broader patient populations. The variability in underlying pathologies and treatment responses may also complicate the interpretation of results, making it difficult to draw broad conclusions about the overall efficacy of MRS and MRP in all patient cohorts. Additionally, while MRS and MRP provide supplementary information, their widespread adoption is limited by the high costs, specialized equipment, and expertise required, making them less accessible in resource-limited settings. Moreover, the interpretation of MRS and MRP data is highly dependent on the experience of the interpreting radiologist, introducing potential variability in results.

Finally, our study focused on patients with inconclusive MRI findings, introducing potential selection bias that may overestimate the utility of these advanced techniques. Although MRS and MRP provide valuable information, they cannot replace histopathological evaluation entirely, and there remains uncertainty in their ability to distinguish between certain conditions, such as different tumor types or differentiating between recurrent tumors and radiation necrosis. Larger studies with more diverse patient populations, longer follow-up periods, and control groups are needed to validate and refine the role of MRS and MRP in neuro-oncology diagnostics and treatment planning.

## Conclusions

Our study highlights the significant potential of MRS and MRP in improving diagnostic accuracy and treatment planning in neuro-oncology, particularly in the Philippines, where these advanced imaging techniques are not yet widely utilized. As the first study of its kind in the country, it paves the way for incorporating MRS and MRP into routine clinical practice, especially in complex cases where conventional MRI alone may be inconclusive. By reducing the number of differential diagnoses, MRS and MRP offered a more definitive imaging diagnosis, enabling clinicians to make more accurate and timely therapeutic decisions. Our findings show that MRS and MRP are helpful in distinguishing between recurrent tumors and radiation necrosis, aiding better management of post-treatment patients. The integration of these modalities with conventional MRI provided a comprehensive multiparametric approach that allowed for the early detection of treatment response or disease progression, which is critical in guiding treatment decisions, particularly in inoperable or malignant midline brain lesions. Additionally, these advanced imaging tools helped in personalizing treatment plans based on each patient's specific condition, further optimizing outcomes.

Despite some limitations, including the small sample size and potential resource constraints, the study highlights the promising role of MRS and MRP in neuro-oncological diagnostics. Further research with larger cohorts and more diverse patient populations is essential to validate these findings and refine the clinical application of MRS and MRP in the management of brain tumors. The results of this pioneering study not only contribute to the advancement of neuro-oncology in the Philippines but also support the broader potential of these imaging techniques in improving patient care worldwide.
